# Radical freedom: Periyar and women

**DOI:** 10.12688/openreseurope.13131.1

**Published:** 2021-03-24

**Authors:** Karthick Ram Manoharan

**Affiliations:** 1 University of Wolverhampton, Wolverhampton, UK

**Keywords:** Periyar, Firestone, Sexual Freedom, Women, Gender, Caste, South India

## Abstract

This paper looks at South Indian social reformer and anti-caste radical Periyar E.V. Ramasamy's approach to the women's question. Periyar was not just an advocate of social and economic equality between the sexes but espoused a radical concept of sexual freedom for women, which is central to his concept of liberty as such. While the anti-colonialists of his period defended native traditions and customs, Periyar welcomed modernity and saw it laden with possibilities for the emancipation of women. Likewise, where other social reformers addressed the women's question within the ambit of the nation and/or the family, Periyar saw both nation and family as institutions that limited the liberties of women. This paper compares his thoughts with *The Dialectic of Sex,* the key work of the radical feminist Shulamith Firestone, and highlights the similarities in their approach to women's liberation and sexual freedom, especially their critique of child-rearing and child-bearing. It explores Periyar's booklet *Women Enslaved *in detail and engages with lesser known, new primary material of Periyar on the women's question, concluding with a discussion of his perspective of the West.

## Introduction

Periyar E.V. Ramasamy’s (Periyar hereafter) writings on women are at the heart of his commitment to a radical concept of freedom. Periyar (1879–1973) was the founder of the Self-Respect Movement in Tamil Nadu, a key figure of Dravidian politics, and leader of the pressure group Dravidar Kazhagam. He is known most for his atheism and radical critique of religion ^1^ and also for his commitment and contribution to anti-caste thought and politics ^2^. However crucial, perhaps even central, to Periyar’s politics of self-respect was his approach to the women’s question. In this paper, I will discuss how Periyar’s approach to the women’s question was grounded not only in a rights-based discourse, but also in a freedom-based discourse; not just freedom from patriarchy, but also sexual freedom in a radically libertarian sense. Several western feminists of the second and third wave of feminism such as Simone de Beauvoir, Kate Millet, Emma Goldman and others have theorized about sexual freedom. I was however particularly intrigued by the work of Shulamith Firestone, *The Dialectic of Sex,* which has been hailed by many as one of the most important works of Western feminist scholarship. What I found striking was the similarity in the diagnosis of women’s oppression and the prognosis for a more gender-just future between Firestone and Periyar. While there is no evidence that either of these two thinkers were aware of the other’s work, it is worthwhile to pursue a comparative reading to place the works of the lesser known and marginalized Periyar in global debates on sexual freedom.

Scholars engaged with feminist politics have looked at the critical importance given to the women’s question and gender in the Self-Respect Movement. In their readings on gender politics in India, Anandhi and Velayutham have highlighted the “limitations in theory itself in dealing with diversities and subalternity” and argue that in a scenario where gender intersects with caste and class, the theory and methods used “should generate knowledge from the margins ^3^.” While feminist scholars like Uma Chakravarti and Sharmila Rege have discussed the intersections of caste and patriarchy, others who have studied the Periyarist politics of gender like Anandhi, Geetha, and Hodges have meticulously captured subaltern perspectives and have made important contributions to the study of women’s politics of and from the margins of Tamil Nadu.

In her 1991 essay “Women’s Question in the Dravidian Movement”, Anandhi provides a concise account of Periyar’s ideas regarding women, perhaps the first of its kind in English-speaking academia, and records the participation and reception of women in the Self-Respect Movement, and the eventual dilution of the radical politics of Periyar once Dravidian political parties became formalized. She says “According to Periyar, while marriage and chastity were key patriarchal institutions, patriarchy as such was ubiquitous, pervading spheres like language, literature and gender-based socialization ^4^.” Geetha, who writes about the resistance of Periyar’s thoughts to quotidian politics, argues that “experiences of the Self-Respect Movement help in theorising the position of those feminists who are critical of and do not wish to ground identity in family and community, and who look to a comradeship to root a new and radical female subjectivity ^5^.” Hodges provides an account of how the Self-Respect Movement revolutionized to some extent ideas on family in 20 ^th^ Century Tamil Nadu, arguing that apart from its activism, “the Self Respect movement also based its campaign for transforming society at a key site of its production: the family and its domestic spaces ^6^.” There is a consensus among these three scholars that family was a main target of critique by Periyar and his followers. In other words, Periyar politicized the personal.

In her paper that looks at caste and masculinities, largely focusing on the non-Brahmin and Dalit movement in Maharashtra, Anupama Rao argues that in the first few decades of the 20 ^th^ Century, non-brahmin critiques of the gender dimension of caste were muted and “caste masculinity was prioritised over the equality of gender” and further that “the subject of non-brahmin and Dalit politics came to be imagined as male ^7^.” The allegation that anti-caste movements did not pay due attention to the gender question has been laid by a few other scholars. In the case of Tamil Nadu, one could say that the movement for non-Brahmin empowerment led by the Justice Party, the precursor to Periyar’s movement, and later by the electoral Dravidian parties, the successors to Periyar, did not have the women’s question as a key component of their discourse. However, to Periyar, gender equality and the freedom of women was a central component in his vision of an egalitarian society. 

This paper builds on the work produced by these feminist academics, but it also taps into recently published works of Periyar on the women’s question. For long, the primary sources for scholars seeking to understand Periyar’s gender politics were the three volumes of Periyar’s selected works published by V. Anaimuthu and Periyar’s booklet *Pen Yen Adimaiyaanal?* (Why was woman enslaved?). I will be referring to G. Aloysius’ translation of the latter, titled *Woman Enslaved,* and the five volumes on *Pennurimai* (Women’s Rights) that were published as a part of Periyar’s collected works in the *Periyar Kalanjiyam.* The works compiled in the first two volumes were published earlier in 1981 and 1991, while the last three volumes were published in 2007. Together, they comprise 1349 pages of Periyar’s writings on women and cover a period from 1926–1973, providing new material that could aid in arriving at a more comprehensive picture of Periyar’s gender politics.

## Gender and caste

In India, sex and caste are so deeply intertwined that it is hard to analyze one without considering the other. The politics of sex, i.e. gender politics, and the sociology of sexual choice, especially with reference to marriage, are colored by caste ^8^. Even in contemporary India, it is not uncommon to see popular matrimonial sites advertising for brides and grooms based on caste. The institution of arranged marriage in India is based on caste. Apart from a few liberal spaces, transgression of caste endogamy is generally frowned upon, at times resulting in violence. Individuals who defy caste norms to love and marry a person from a different community have faced social ostracism, physical assault, even murder. Commonly called ‘honor killings’, these acts of violence usually occur when the woman of a higher caste falls in love or elopes with a man of a lower caste, though the other way around also happens at less frequency. ‘Honor’ as viewed by caste society, rests in regulating the sexuality of women and maintaining their caste purity ^9^. In these honor killings either the couple, or the individual man or woman held responsible for violating the honor of the community is killed. Several such cases have been reported in India, in developed and underdeveloped states alike, cutting across several communities. Not just that, even intermarriages between Dalit castes, such as the Paraiyars and the Arunthathiyars in Tamil Nadu, or the Malas and Madigas in Andhra Pradesh, have been discouraged at a community level ^10^. A particularly gruesome case of honor killing in recent times was that of Kausalya-Sankar in Tamil Nadu.

Kausalya who hailed from an economically backward but politically significant intermediate caste group, the Thevars, fell in love with Sankar, a Dalit youth, in 2014. Despite much opposition from her family, which included her being beaten and locked up and Sankar being threatened with death, the couple defied caste norms and married in 2015 and fled to another town. Within a year, henchmen hired by Kausalya’s father tracked the couple down and attacked them brutally in broad daylight, leaving Sankar dead and Kausalya grievously wounded. This incident, captured on CCTV, became the subject of an Al Jazeera documentary on honor killings. Kausalya’s father and his henchmen were eventually sentenced to death by a court. Her relatives however believe that she was in the wrong to transgress the laws of caste and marry an outsider.

Dalit castes are also not immune to committing violent acts to punish transgressions of caste endogamy. For instance, in 2003, an Arunthathiyar woman from Vilupuram was raped and murdered because of her brother’s marriage to a Paraiyar woman. Commenting on such incidents, G. Jakkaiyan, a prominent Arunthathiyar leader observed that “a Pallar household found it uncomfortable to accept any marital connection with Parayars, while Parayars and Pallars in turn shied away from marital alliances with Arunthathiyars”, adding that “Almost all castes in Tamil Nadu have been Brahminised. And Dalits are no exception ^11^.” It is not surprising that upper castes would have considerable anxieties regarding intercaste marriages. But why would economically backward castes like the Thevars, who for a period of time were categorized as criminal tribes by the colonial regime, and even Dalit castes subscribe to the idea of caste endogamy?

Ambedkar described women “as gateways of the caste system” and Rege calls this an “unprecedented sociological observation ^12^.” The historian Suvira Jaiswal argues that endogamy *alone* is not the root of the caste system, and it was “the emergence of a class society in which patriarchal control played an important role in securing the rights and privileges of the elite on a hereditary basis ^13^.” Endogamy was not necessarily the cause, but definitely a central component of the caste system, with strict punishments prescribed to transgressors. Jaiswal identifies two key features of the caste system – “the subordination of women” and “its capacity to reinvent itself in changing social formations in the service of the powerful and the dominant ^14^.” For the dominant castes, guarding the chastity of their women was important to preserving the purity of their caste and to prevent the mixing of varnas, i.e., *varnasamkara ^15^.*

It is worth considering in some detail Uma Chakravarti’s hugely significant work *Gendering Caste.* Chakravarti argues that “Each caste is a closed and bounded group, and all social relations are represented in terms of bounded groups” – crucially, the relation of marriage ^16^. The notion of purity of the socially powerful castes made them try to posit it as the universal hierarchical principle and claim that it had the consent of all the castes. She argues that to understand the intersection of caste and gender in India, it is important to consider how reproduction is organized, who controls female sexuality, and the ideologies that sanction this ^17^. Caste needs endogamy to reproduce itself as a system and endogamy is a tool for both caste and gender subordination. Endogamy, she argues, “is a necessary feature of a society *stratified on the basis of birth,* as different strata would not be able to maintain their distinctive identities without it ^18^.” Chakravarti then gives a succinct definition of the ideology that links caste and gender, making them interdependent and prescribing rules to govern society.

“brahminical patriarchy… is a structure unique to Hinduism and the caste order… It is a set of rules and institutions in which caste and gender are linked, each shaping the other and where women are crucial in maintaining the boundaries between castes ^19^.”

Brahminical patriarchy, though designed by the Brahmins, was successful only because it was internalized by the majority of non-Brahmins the result being that lower castes also suppress female sexuality “without being *conscious* that these norms are derived from the very *structures* that *oppress* them in other ways ^20^.” Thus, not only the upper castes, but the backward castes like Thevars or Dalit castes like the Paraiyars also internalize Brahminical patriarchy when they place restrictions on the movements of ‘their women’, where the community dictates who to marry and who not to.

Two important concepts of Brahminical patriarchy were *pativrata* (devotion to husband) and *stridharma* (duties of a woman). Brahminical law-givers like Manu and Yajnavalkya, not to mention the Hindu epics and puranas, provide detailed prescriptions of rules and regulations for the high-caste woman based on these concepts. The ancient texts feared the *strisvabhava*, the essential nature of woman, and believed that it had to be tamed by *stridharma* ^21^. However, these prescriptions were largely for the high-caste woman, while the lower caste women were seen as loose, immoral, and sexually available for upper-caste men. The practical result of this was that any caste which seeks social mobility within the caste system needed to adopt *stridharma* and place stronger restrictions on its women, their movement and their choices.

As we shall see in the following sections, Periyar called on women of all castes to break not just notions of chastity and honor, but all forms of community-based restrictions; not just to fight caste, but also equally to emerge as free individuals.

## The dialectic of sex

It should be stated upfront that *The Dialectic of Sex* makes several reductive observations on thinkers like Marx and Freud, and it has also been criticized by others for its approach to race. Yet, this work has been deemed important to feminist thought because it firmly grounds sexual freedom, especially freedom from both childrearing and childbearing, as an important component of the liberation of all women. Firestone believed that the fight against sexism was one of the toughest because it was not only Western culture, but all of culture as well as nature itself that had to be fought. To Firestone, it was not just the economic division of sexual labor, but the very biological “fact” of sexual difference was oppressive and she called the heterosexual biological family “an inherently unequal power distribution ^22^.” Firestone did not consider what is natural or biological to be a just human value, and argued that sexual discrimination could not be justified by citing its origins in the natural world. The problem of the women’s question was political, not biological, and Firestone called for a “seizure of control of *reproduction*” and “not just the elimination of male *privilege* but of the sex *distinction* itself ^23^.” A revolution that sought to end all exploitation must first uproot the biological family because to Firestone, “The heart of women’s oppression is her childbearing and childrearing roles ^24^.”

Firestone believed that matriarchy could never be an answer to women’s oppression and she dismissed the worship of female/mother goddesses. “To be worshipped is not freedom. For worship still takes place in someone else’s head, and that head belongs to Man ^25^.” Dependence on men was the origin of inequality for women, and self-regulation was the basis of freedom ^26^. A key hindrance to the self-regulation and self-determination of women was pregnancy. Firestone asserted that “Pregnancy is barbaric” and called for women to protest the role of motherhood imposed on them by nature and culture ^27^. Women were disadvantaged materially, emotionally, psychologically and culturally because of child-birth, which made them a “slave-class” utilized for the reproduction of the species ^28^.

To Firestone, the first demand for a new system should be “The freeing of women from the tyranny of their reproductive biology by every means available, and the diffusion of the childbearing and childrearing role to the society as a whole, men as well as women ^29^.” She supported family planning measures and communally run day-care centers as a temporary step for increasing the freedom of women. Ultimately, artificial reproduction must be brought in as a replacement for biological conception, so as to “free women from their biology ^30^.” She imagines a cybernetic socialism where childbearing will be taken over by technology, thereby widening the freedoms of women. Feminism, as Firestone saw it, was “the inevitable female response to the development of a technology capable of freeing women from the tyranny of their sexual-reproductive roles ^31^.” The spirit of this technology-supported feminism was not just about having equality with men, but freedom from patriarchy and other forms of oppression, and freedom towards a radical self-determination.

It is quite remarkable that Periyar, a male who was born in a socially conservative background in the family of an intermediate trading caste and operated in a patriarchal socio-historical context of a ‘Third World’ country, should develop a strikingly similar radical position as regards the women’s question almost 50 years before Firestone. Like his approach to atheism, discussed in detail in an earlier paper ^32^, it is hard to identify a singular source of inspiration for Periyar’s gender politics. He was not only too radical for his contemporaries, but also his successors, who either diluted or plainly ignored his gender politics. This would seem rather ironical, given that E.V. Ramasamy was formally given the title ‘Periyar’ in 1938 at the Tamil Nadu Women’s Conference where among others, Annai Meenambal Sivaraj, a prominent Dalit woman leader, was a key participant. While Periyar is considered as the *Thanthai,* or Father, of the Dravidian Movement, the feminist concerns of this father figure are often brushed aside in popular public discussions, but for the rare academic or activist concerned with gender justice. This omission does this thinker much injustice because, as I seek to argue, his conception of freedom cannot be grasped in its totality without engaging with his ideas on the women’s question.

## Women enslaved

From his entry into political activism in the 1920s, Periyar was acutely conscious of involving women in politics and social reform, not as mere subordinates, but as comrades. In the Vaikom agitations, he encouraged the mass participation of women, and his wife Nagammai played an important role in the agitations too. He recognized that his peers in the Congress party and the Justice Party did not give adequate importance to the women’s question and at times, sought to sideline it. To Periyar, however, women’s liberation was a key to ending oppression in society. In the Chengalpet Self-Respect conference held on 17–18 February 1929, Periyar and the Self-Respecters passed resolutions demanding equal rights of property for women, widow remarriage, abolition of child marriage, freedom to choose spouses defying caste and community norms, besides encouraging women to enter professions of their choice. These resolutions rattled not just the Congress, but also many in the Justice Party, and thus Anandhi rightly notes that “Periyar's commitment to the cause of women's emancipation often led him to be critical of his own political comrades” and he often challenged the Justice Party on their approach to the women’s question ^33^. The articles that comprise *Women Enslaved* were written in this context.

*Women Enslaved* constitutes what can be called Periyar’s ‘early writings’, and is comprised of ten essays he wrote on the women’s question between 1926 and 1931. These essays were compiled and published as a booklet in 1934, perhaps the first work of Periyar to be published in a book form, under the title *Pen Yen Adimaiyaanal?* This booklet provides a general introduction to Periyar’s approach to the women’s question, his analysis of the cause of their oppression and his suggestions for change, and has been an important reference on the subject. In his preface to a 1942 reprint, Periyar wrote that the aim of the booklet was to “demonstrate the different factors by which women were enslaved and continue to be so enslaved, and also the different ways and means by which they could emancipate themselves and live as free people”, adding that this booklet was written for both men and women, and for “people of all religions, societies and nations” ^34^. One may be tempted to draw parallels between this work and Frederick Engels’ *The Origins of Family, Private Property and State,* but where Engels locates the oppression of women in political economy, Periyar locates it in culture, primarily the ‘Aryan’ and brahminical, but he did not spare Tamil culture either.

Periyar challenged the value that conservative Tamil society placed on *karpu,* which can be loosely translated as chastity, as a social hypocrisy that limits the freedom of women while placing no such limits on men. He wondered why this is the case, and accused both Hindu religious texts and Tamil secular texts like the *Thirukkural* for engendering oppression. That Hindu religious and moral texts prescribed rigid and caste-based notions of chastity is well-known now. Chakravarti notes that “ *pativrata* the specific dharma of the Hindu wife then became the ideology by which women accepted and even aspired to chastity and wifely fidelity as the highest expression of their selfhood ^35^.” Ancient Tamil poets like Thiruvalluvar, Ilanko Adigal and Auvaiyar also eulogized the virtues of chastity.

To the Tamil nationalists of Periyar’s time, these ancient poets were not only central to their modern political imagination, but they were also invoked to oppose Aryan-Hindi-brahminical supremacy. The fervent ideologues of Tamil nationalism projected the notion of chastity and purity onto a secularized, but desexualized, *Tamil thaai* (Mother Tamil), who stood for a feminized pure, chaste, virgin Tamil language to be protected from a corrupting outside influence ^36^. While Periyar was unreservedly critical of Brahminism and Hindi imposition, he was an uncomfortable presence for the Tamil nationalists. Not only did he mock the romanticization of the Tamil language, he also did not excuse the classical Tamil poets, thinkers and saints for what he thought were their condescending views on women. He condemned Thiruvalluvar for promoting “ideas of slavishness” and asked if the poet would have written the same thing had he been a woman ^37^. Arguing that the *Thirukkural “*does not advocate uniform morality for both the sexes”, Periyar says that morality and chastity, if they are valid at all, should be seen as equal for both women and men ^38^. Periyar, who saw chastity as a device of oppression coded by religion, ultimately wanted women to be liberated from the clutches of this concept. It is of interest to note that around the same time, Virginia Woolf wrote in her *A Room of One’s Own* that “Chastity had then, it has even now, a religious importance in a woman’s life, and has so wrapped itself round with nerves and instincts that to cut it free and bring it to the light of day demands courage of the rarest ^39^.” In colonial Tamil Nadu, Periyar perhaps could be credited for demonstrating that rare courage.

Periyar also recognized the pervasiveness of patriarchal ideology and notes that the concept of chastity makes women complicit in their own oppression. And though he spoke sympathetically of Islam in other places, in this booklet he condemns the practice of veiling and polygamy that restrict the movement of women while men can have multiple partners ^40^. He argued that “If women are to be truly liberated this gender-biased and enforced practice of chastity is to be abolished and in its place gender-neutral, egalitarian and voluntary practice of chastity is to be established”, calling for the abolition of marriages, laws, and religions that force women to remain subservient to men ^41^.

Periyar proceeded to attack the idea of ‘love’ as has been traditionally understood. It is notable here that ideal forms of love were greatly eulogized in ancient Sangam Tamil poetry. For instance, the *Ainkurunuru,* an anthology compiled roughly in the second to third centuries AD, consists of five hundred poems dedicated exclusively to love. What is significant about this poetry is that it is located in the secular, where gods rarely figure at all. Again, while such poetry was celebrated by the Tamil nationalists, Periyar dismissed them for providing rigid, essentialized notions of the genders. Periyar revolted against the ancient, and he found the ancient revolting. He believed that ancient notions of love and undying commitments had no place in a rational, modern society. He calls-out such advocates of love to be “ignorant of general natural dispositions” ^42^. He believed that biological callings and the freedom to choose should be criteria for partnership, not culture or pressures from family or society. He argues that the idealization of love or the family relationship would force couples to live in “perennial dissatisfaction and harassment ^43^.” Instead of idealizing love, Periyar sought to view it in a utilitarian way, as a private emotion that should contribute to the wellness and pleasure of individuals and not a norm imposed by society that compels people to remain bonded in unjust relationships. Periyar opposed the idea that “people’s expressions of love and desire must be brought within certain discipline and they ought to be expressed between particular individuals, in a particular way only ^44^.” In other words, he felt that disciplined and idealized notions of love chained women to their oppression and caste.

If love is attacked, marriage cannot be far behind. Periyar said that the philosophy of marriage in India is an atrocity committed on women, “which leads to the enslavement of women by men ^45^.” Again, he believed that idea of the sacredness of marriage is but part of the patriarchal ideology to cheat women into accepting their own enslavement. He believed that across the world, marriage functions as a cruel institution; but while the conditions have relaxed in the West, especially in Soviet Union thanks to the socialist revolution, India “is doggedly holding on to the same old practices ^46^.” He argued that legal provisions to annul marriages and approve of remarriages alone can guarantee the freedom of men and women. He said that the progress of women is not possible without creating the space for the cancellation of marriages ^47^. He viewed marriage as a “contractual agreement entered into by a couple for the purpose of their own life-comfort ^48^.”

Periyar viewed love and sexual desire as fundamental freedoms that should not be regulated by social and cultural institutions, and advocated that the freedoms that exist for men should exist for women as well. However, prohibitions against adultery, and the social derogation of women who had more than one sex partner, were values that sought to force women to conform to the notions of chastity. Periyar noted how a woman who had multiple sex partners was derogatorily called a *vibachaari* (adulteress/prostitute) but a man who did the same was not called a *vibachaaran* (adulterer/gigolo) ^49^. He observed how the notion of the purity of the women is closely connected to the purity of the caste and clan, a result of which the ‘adulteress’ is thrown out of the caste and ostracized, while the philandering man takes pride in his conquests.

Periyar believed that India would not attain full freedom without women and the untouchable castes attaining their freedoms and equal rights. Without foregrounding this, he argued, “to handover the responsibility of such depressed people’s freedom and welfare to us is equivalent to handing over the goats to the butcher, and not otherwise ^50^.” Women and the untouchable castes needed to understand the true meaning of liberty and freedom for themselves and not rely on others to give it to them. He said that it is evident from the manner they treat women and the untouchables that Indians have no sense of freedom at all. One step towards enhancing the freedom of women was to ensure that they had an equal right to hereditary property, educational training and economic independence ^51^. The other was contraception.

Periyar noted that contraception had been advocated by others for the sake of women’s or children’s health or for maintaining a small family, but the Self-Respect movement wanted to advocate it for “liberation and autonomy of women ^52^.” He saw pregnancy as “positively harmful to the autonomy of women” and blamed it for being the root cause of “women’s frequent illnesses, aging before time and premature death ^53^.” Women, if they are to enjoy freedom, should stop bearing children. Contraception, thus, was not to be advocated only for health reasons, but also because it contributed to the freedom of women. He argued that there must be a wide propaganda in favor of contraception through books, theatre and cinema. While Periyar argued that the absence of childrearing and childbearing responsibilities would enhance the freedom of both men and women, he firmly believed that only women could be the agents of their own liberation. Rhetorically he asked “Could goats and chickens anywhere be freed by wolves?” adding that just as Brahmins would not liberate non-Brahmins, neither would men liberate women. 

The booklet concludes with the assertion that “masculinity” must be destroyed if women are to be liberated. *Aanmai* which is the corresponding Tamil word for masculinity, is generally seen as a positive term that also denotes virility, courage and straightforwardness in a man. On the other hand, *penmai,* or femininity, is associated with docility and purity of character and body. Periyar argued that it is the concept of masculinity that is socially and culturally constructed so as to elevate men at the expense of women, and thus, without demolishing the idea of masculinity and the virtues associated with it, the freedom of women would not be possible. Further, he said that masculinity prescribes certain oppressive roles to femininity, most notably that of motherhood, often by appeals to biological nature. Periyar however rejects this appeal to biological nature saying that to be human is to be “against nature” and that one should not worry if as a result of the abolition of childbearing, humanity does not expand – according to him, it was an unjust argument to speak of the propagation of humanity at the expense of women ^54^.

We can see here that Periyar anticipates Firestone’s arguments by almost 50 years. Like Firestone, he locates the oppression of women to (what a male-dominated society relegates as) their biological functions, namely the bearing and rearing of children. Noting that masculine norms impose unfair standards of love and chastity through institutions like family and marriage on women, he argues that these need to be discarded for the creation of a truly egalitarian society. Finally, he advocates freedom in its most radical sense, as both a means and an end, for women.

## The centrality of women’s freedom

Periyar called the conventional marriage system, which requires the woman to stay with the man even if there is mutual incompatibility, a form of slavery ^55^. In particular, he opposed the brahminical rituals in marriage and he saw Hindu customs and practices as evidence of oppression of women. He saw the traditional marriage as an institution that maintained caste purity. He argued that marriages should happen without the influence of caste, religion, god, traditions and rituals, as these practices legitimized the enslavement of woman to man ^56^. Accepting brahminical marriage rituals not only degraded women, but also non-brahmin men as they consented to being inferior to the priests officiating such rituals ^57^. As an alternative, he promoted self-respect marriages based on friendship, equality and respect. A precedent to this was the Satyashodak marriages advocated by Maharashtrian anti-caste radicals, which were conducted without brahmin priests and emphasized equality and self-respect between the married couples. Rao argues that both these forms of marriages “constituted an explicit challenge to the social reproduction of caste … through the sexual regulation of women ^58^.” Complementing this, Geetha says

“[…] the Self-Respecters sought to reclaim love and desire from within the matrix of the Hindu marriage sacrament and invest these with a liberative significance. This reclaiming was complex and somewhat problematic, for though Self-Respecters succeeded in constituting love and desire as natural, self-validating emotions, they yet felt obliged to restore to a social value, though of a radically different kind. In fact the institution of a Self-Respect marriage ritual was part of this attempt to grant a released and potentially anarchic emotion a coherent and viable embodiment ^59^.”

There were Hindu organizations like the Arya Samaj that also conducted inter-caste love marriages, which they continue to do till date. But Dhanda observes that in these marriages, “the orthodoxy of the Hindu priest is not itself challenged” and that they did not aim to provide alternative, humanist marriage practices ^60^. On the other hand, the fundamental requirement for a self-respect marriage was the absence of the Hindu priest and rituals. Periyar wanted self-respect marriages to be based on a secular contract, as a form of companionship, that can and should be called off if any of the partners so desired. He believed that this freedom to walk out of an unhappy – or even just boring – marriage was important to women’s freedom. He also believed that marriage would become unnecessary in the future. As if anticipating contemporary debates on post-humanism, in 1961 he speculated a future where ‘human society’, with its biological family bonding, would cease to exist, and machines would play an increasingly important role ^61^. Like Firestone, he does not want women to be determined by nature or biological functions, but looks towards technology for providing the tools of the liberation of women. 

Periyar rejected looking to the past for moral instruction in the present. “We are new humans. Our ancestors were barbarians. They held barbaric opinions ^62^.” He felt that appeals to the past were excuses for oppressive practices in the present. In the 60s, at a time when the figure of Kannagi, the key character of the Tamil epic-poem *Silappathigaaram* was celebrated as a symbol of Tamil civilization, he called the work a “dustbin” that enforces ridiculous concepts of chastity on women and called on the thinking public to not pay heed to such literature ^63^. He said that classic literature eulogized the beauty of women only so that they would remain objects of sexual fantasy for men, and thus, urged women to stop beautifying themselves and focus instead on scientific learning ^64^. He made such suggestions at several wedding functions where he was invited to preside over. He also argued for women to take over 50% of the jobs so as to ensure their economic independence ^65^. He was of the opinion that women should not worry about femininity and be represented in all spheres of social life, and stated “If I could establish dictatorial rule, the first thing I would do is to destroy the discrimination of women and the preferential treatment of men ^66^.”

At an Adi Dravida meeting, Periyar said that the oppression of women is worse than the oppression of the ‘lower’ castes, workers and peasants ^67^. He criticized the inherent hypocrisy in the sexual economy in India where a man could have multiple sexual partners but a woman is forbidden from pursuing her desires. He attacked the customs that wanted the woman to repress their sexual pleasure. He said that women should also have the freedom to have multiple sexual partners like men. He refused the logic of critics who said that it would be better to preach morality to men than immorality to women. “Much advice has been given to men over the ages. Marriage has become an institution of slavery. No man is a saint ^68^.” He called on women to beat back men who physically abused them and verbally abuse those who verbally abused them. He said that if a marriage could not recognize this freedom to retaliate, then it should be dissolved. Further, highlighting the sexual freedoms in the west, he said that married women should be given the freedom to choose other sexual partners and walk out of a failed marriage without fearing legal or social punishment ^69^.

Periyar said that a married woman is treated as an unpaid wage slave. “A wife is an unpaid servant for a man. Even a servant cannot be beaten or kicked around. But a wife can be beaten or kicked and others will not interfere ^70^.” He saw the oppression of women operating in dual ways; one economic, which treated them as unpaid laborers at home and thrust upon with the burden of housework including the bearing and rearing of children; the other cultural, the moral codes of Brahminism, which legitimized their oppression, glorified it, and made them complicit in it. He said that for 3000 years, oppression of women went unchallenged because “men thought it right to give respect and pay obeisance to Brahminism ^71^.” Brahminism provided a system of values to justify patriarchy and the degraded position of women and, as Uma Chakravarti puts it, “ *brahmana women must consent to brahmana ideology for it to be effective ^72^.*” Periyar says it more bluntly “In this country whichever woman remained a foolish slave, those women were called as *pativratas* by the Brahmins ^73^.” Periyar, of course, was not just talking about Brahmin women here, but women as such. He believed that Brahminism and patriarchy interacted in convenient ways to restrict the liberties of women in general. So for instance, he called the Tamil Jaina text *Silapathigaram,* his criticisms of which we have discussed in brief above, as a work created by “rummaging through Brahmin garbage ^74^.” While recognizing Brahminism as a fundamental doctrine of inequality, he was also unsparing towards Tamil patriarchal traditions, which he thought collaborated with Brahminism to create a native system of gender inequality. Which is why one of his key aims was not just to ‘denationalize the past’ ^75^, but also to ‘denationalize’ the bodies of women. 

## Conclusion: looking west from the south

In her most celebrated essay ‘Can the Subaltern Speak?’, Gayatri Spivak writes that “Imperialism's image as the establisher of the good society is marked by the espousal of the woman as object of protection from her own kind ^76^.” Spivak gives the example of Sati or the Hindu practice of burning widows on the pyre of their dead husbands, and explains how the colonial intervention into the banning of this practice was done with the intention of legitimizing the colonial rule and also silenced the voice of the ‘victim’ they were saving. Periyar, however, thanked the “white man” without whom, “in the name of religion and tradition, women would have been burnt along with their dead husbands. It was only the white man’s law that changed this ^77^.” Periyar had no faith in the Indian nationalist project or in local Tamil nationalist imaginations to secure liberation for women. If anything, he viewed them as movements that reinforced patriarchy and misogyny, which he argued, were inherent to Hindu society.

Partha Chatterjee argues that from the 19 ^th^ Century onwards, Indian nationalism located the women’s question in the inner domain of sovereignty: “The inner domain of national culture was constituted in the light of the discovery of tradition ^78^.” Indian nationalism, Chatterjee says, saw the women’s question as a problem of Indian tradition, i.e. something meant to be resolved internally. Indian nationalist reason made an inner/outer distinction, where the outer was the world, which was material and political, while the inner was the home, which was spiritual and true. And central to the inner domain was the woman. In protecting the inner, nationalist reason wanted to protect the spiritual essence of national culture, while approaching modernity selectively ^79^. The responsibility fell on women to nurture spirituality at home and her freedom could be found only in her contribution to the building of a superior national culture. On the other hand, the westernized woman, as different from the “normal” Hindu woman, was seen as “brazen, avaricious, irreligious, sexually promiscuous ^80^.” Further, since Indian nationalism “located its own subjectivity in the spiritual domain of culture, where it considered itself superior to the West and hence undominated and sovereign”, nationalist reformers did not want the colonial state to intervene through legislations in the realm of Indian culture and they did not want to negotiate with the colonial state on the women’s question ^81^.

For instance, Indian nativists strongly opposed the Hindu Widow Remarriage Act (1856), Age of Consent Bill (1861), and the Act III of 1872 that introduced civil marriage to India. In independent India, upper-castes also opposed the Madras Devadasis (Prevention of Dedication) Act which became a law in October 1947. The opponents of such legislations, claiming to guard tradition, did not believe in the agency of individuals to act – only the community had agency to decide, and duty towards the community took precedence over individual rights ^82^. Geetha also notes that the identification of the interior as the location of pure culture and tradition, which is not to be contaminated by external influences, operated not just in national community, but the caste community as well ^83^. One could advance the thesis, which will require a deeper analysis than possible in this paper, that underlying the imagination of the national community was the psychology of the caste community which sought to protect the pure inner from the impure outer.

Periyar welcomed the liberating effect some aspects of colonialism had on women in the Tamil society and viewed westernization positively. He found the native national culture and its effects on women to be harmful and he believed that the arrival of Western modernity and the spread of English education contributed to the wider reach of modern egalitarian values with respect to the women’s question. He asserted that the ‘white man’ banning Sati and providing women access to modern education was one of the reasons for the anti-colonial movement which wanted to preserve traditional, reactionary values. In Periyar’s perspective, the Brahminical culture, the influence of religion, and Tamil patriarchy all contributed to the oppression of women and women were better off westernized than relying on native cultural-social values for liberation. Periyar condemned native or traditional dresses like the saree and the Islamic veil ^84^ as regressive, and asked women to adopt Western attire to attain greater mobility.

While Periyar mostly viewed the situation of women in European countries as better than in India, he also felt that patriarchy, which was only superficially challenged under capitalism, nevertheless constrained the full freedoms of women in these countries. Likewise, while he viewed the achievements of the Soviet Revolution positively as regards to women empowerment, he did not have faith in the Indian communists to work for the liberation of the marginalized sections. Periyar saw both the European and the Soviet models of women’s mobility as inspiration for women here, but he also wanted the women’s question to be addressed as its own, autonomous, socio-political issue and not to be subsumed by nationalist, Dravidianist, or the anti-caste discourse. This is not to say that he saw gender as uninformed by nation, region, class or caste; only that women from these communities must exercise their full autonomy and realize their full freedoms – social, economic and sexual. 

The practical effects of Periyar’s discourse in his time could be seen in that “The women members of the Self Respect Movement not only participated in the non-agitational programmes of the movement like conferences, but also quite actively in mass agitations ^85^.” Not just that, women from the most marginalized sections, like that of the devadasis or the ‘temple prostitute’ communities, also became active participants in social reform movements in the state. Women from non-Brahmin subaltern communities took part in anti-caste struggles, the anti-Hindi agitations and protests demanding greater social and economic rights for women. As mentioned before, Periyar’s approach to gender was too radical for his contemporaries and his successors. In fact, a hetero-patriarchal understanding is a commonality among the major Dravidian political parties in the state. References to women as the bearers of Tamil culture and carers of the Tamil children of the future are common. Likewise, it would require a whole new essay to look at the routine sexism in popular Tamil cinema. While Periyar is often invoked by several anti-caste Dravidian and Dalit leaders, rarely is he invoked as a feminist, except by a few scholars and activists who are committed to recording his contributions to the women’s question.

However, the emergence of new publics and new voices from the margins may perhaps rekindle an interest in the radical potential of Periyar’s gender politics. Kausalya, mentioned above, became an activist immediately after Sankar’s death. She was the key witness against his murderers and she worked with Periyarist and Dalit organizations to bring them to book. She continues to work with these organizations to oppose casteism and honor killings. In 2018, she married a folk artist in a Periyarist self-respect marriage. In a speech in 2017, she pledged herself to the cause of social justice, annihilation of caste, and liberation of Tamils, citing Periyar as an inspiration. She asserted “Without the slightest hesitation, I should break the cultural and social chains that bind me as a woman, and liberate myself. Thanthai (father) Periyar taught me to do that ^86^.”

Likewise, the reception of Periyar’s gender politics among transpersons in the state is an area that needs to be studied further. Living Smile Vidya, a prominent Queer rights activist and performance artist from Tamil Nadu, proudly declares that she is a Periyarist. To cite her

“Periyar is someone who spoke about women’s rights. I am more familiar with him than other anti-caste writers and have always considered myself a Periyarist. In fact, Periyar wrote in *Kudi Arasu* in 1928, about how women should give up their reproductive labour and use birth control if they want to be free. In that sense, I feel I am the ideal, liberated woman that Periyar always spoke about ^87^.”

The debates among newly emerging feminists and LGBTQ activists in South India, who are conscious of the intersections of caste, class, different sexualities and gender identities, might contribute to a further enriching of Periyarist thought and inform discussions on sexual freedom and gender equality across the world.

## Data availability

No data are associated with this article.

## Notes

^1^ Karthick Ram Manoharan. 2019. "Freedom from God: Periyar and Religion." *Religions*, 11(1): 10. For Special Issue on *Dalits and Religion: Ambiguity, Tension, Diversity and Vitality.* Eds. Cosimo Zene and Meena Dhanda.

^2^ Karthick Ram Manoharan. 2020. “In the Path of Ambedkar: Periyar and the Dalit Question.” South Asian History and Culture, 11:2. pp. 136-149.

^3^ S. Anandhi and Meera Velayutham. 2010. “Rethinking Feminist Methodologies.” *Economic and Political Weekly*. Vol. 45, No. 44/45. p. 40.

^4^ S. Anandhi. 1991. “Women’s Question in the Dravidian Movement, c. 1925–1948.” *Social Scientist.* Vol. 19, No. 5/6. pp. 26.

^5^ V. Geetha. 1998. “Periyar, Women and an Ethic of Citizenship.” *Economic and Political Weekly*. Vol. 33, No. 17. p. WS15.

^6^ Sarah Hodges. 2005. “Revolutionary family life and the Self Respect movement in Tamil south India, 1926–49.” Contributions to Indian Sociology. 39. p. 257.

^7^ Anupama Rao. 2018. “Caste, Masculinity, and Alternate Genealogies of the Feminist Subject.” In *Gender, Caste and the Imagination of Equality.* Ed. Anupama Rao. New Delhi: Women Unlimited, p. 175.

^8^ See Meena Dhanda. 2012. “Runaway Marriages: A Silent Revolution?” *Economic and Political Weekly.* Vol. 47, No. 43. pp. 100–108, and Sowjanya Tamalapakula. 2019. “The Politics of Inter-caste Marriage among Dalits in India: The Political as Personal.” *Asian Survey.* 59 (2). pp. 315–336.

^9^ In a text originally published in 1916, Ambedkar identifies the centrality of endogamy in maintaining caste as an enclosed community. See B. R. Ambedkar. 1985. *Castes in India: Their Mechanism, Genesis and Development*. Jullundur: Patrika Publications.

^10^ ‘Dalit’ is a term commonly used to refer to members of the untouchable castes in India, who currently come under the administrative category of Scheduled Castes. Despites attempts for unification under the Dalit identity, frictions and conflicts persist between theses castes in various regions of India. In Tamil Nadu for instance, Arunthathiyars routinely complain that the caste discrimination that they face from the Paraiyars is no different from the practices of the dominant castes. The YouTube channel *Dalit Camera* has recorded several such testimonies from Arunthathiyars. Also see Ravichandran Bathran. 2016. “The Many Omissions of a Concept: Discrimination amongst Scheduled Castes.” *Economic and Political Weekly.* Vol. 51. No. 47. pp. 30–34.

^11^ Ilangovan Rajasekaran. 2020. “Purity and Honour.” *Frontline.* 13 March. https://frontline.thehindu.com/cover-story/article30911129.ece [Accessed 12 February 2020]

^12^ Sharmila Rege. 2013. *Against the Madness of Manu: B.R. Ambedkar’s Writings on Brahminical Patriarchy.* New Delhi: Navayana. p. 65.

^13^ Suvira Jaiswal. 2016. *The Making of Brahmanic Hegemony: Studies in Caste, Gender and Vaisnava Theology.* New Delhi: Tulika, p. 5.

^14^ Ibid., p. 58.

^15^ Ibid., p. 118.

^16^ Uma Chakravarti. 2018. *Gendering Caste: Through a Feminist Lens.* New Delhi: Sage, p. 10.

^17^ Ibid., p. 25.

^18^ Ibid., p. 28.

^19^ Ibid., pp. 32–33.

^20^ Ibid., p. 34.

^21^ Ibid., p. 69.

^22^ Shulamith Firestone. 1972. *The Dialectic of Sex: The Case for Feminist Revolution.* New York: Bantam Books. p. 8.

^23^ Ibid. p. 11.

^24^ Ibid. p. 72.

^25^ Ibid. p. 74.

^26^ Ibid. p. 97.

^27^ Ibid. pp. 198–199.

^28^ Ibid. p. 205.

^29^ Ibid. p. 206.

^30^ Ibid. p. 206.

^31^ Ibid. p. 31.

^32^ Manoharan. "Freedom from God."

^33^ Anandhi. “Women’s Question in the Dravidian Movement”, p. 27.

^34^ Periyar E.V. Ramasami. 2009. *Women Enslaved.* Translated by G. Aloysius. New Delhi: Critical Quest. p. 8.

^35^ Uma Chakravarti. 2013. *Conceptualising Brahmanical Patriarchy in Early India: Gender, Caste, Class and State.* New Delhi: Critical Quest. p. 19.

^36^ Sumathi Ramaswamy. 1998. *Passions of the Tongue: Language Devotion in Tamil Nadu, 1891–1970.* New Delhi: Munshiram Manoharlal. p. 114–126.

^37^ Ramasami. *Women Enslaved.* p. 10.

^38^ Ibid., p. 16.

^39^ Virginia Woolf. 2009. *Liberty.* London: Vintage, p. 11.

^40^ Ramasami. *Women Enslaved*. p. 16.

^41^ Ibid., p. 11.

^42^ Ibid., p. 17.

^43^ Ibid., p. 20.

^44^ Ibid., p. 27.

^45^ Ibid., p. 21.

^46^ Ibid., p. 22.

^47^ Ibid., pp. 22-23.

^48^ Ibid., p. 24.

^49^ Ibid., p. 29.

^50^ Ibid., p. 39.

^51^ Ibid., p. 41.

^52^ Ibid., p. 42.

^53^ Ibid., p. 43.

^54^ Ibid., p. 46.

^55^ Periyar E.V. Ramasamy. 2007. *Periyar Kalanjiyam* (22): Pennurimai Pagam 3. Chennai: Periyar Suyamariyadhai Prachara Niruvanam, p. 11.

^56^ Ibid., p. 43.

^57^ Ibid., p. 74.

^58^ Rao. “Caste, Masculinity, and Alternate Genealogies of the Feminist Subject”, p. 174.

^59^ V. Geetha. 2015. *Self-Respect Marriage: A Tale of Self-Respect Unions & What Happened to Them.* New Delhi: Critical Quest, p. 20.

^60^ Meena Dhanda. “Runaway Marriages.” p. 105.

^61^ Ramasamy. *Periyar Kalanjiyam* (22). pp. 39-40.

^62^ Ibid., p. 11.

^63^ Ibid., p. 66.

^64^ Ibid., p. 217.

^65^ Ibid., p. 221.

^66^ Periyar E.V. Ramasamy. 2008. *Periyar Kalanjiyam* (31): *Matham Pagam 7*. Chennai: Periyar Suyamariyadhai Prachara Niruvanam, p. 168.

^67^ Periyar E.V. Ramasamy. 2018. *Namakku Yen Intha Izhinilai? Jaathi Manadugalilim Jaathi Ozhippu Maanadugalilum Periyar*. Ed. V.M. Subagunarajan. Chennai: Kayal-Kavin, p. 177.

^68^ Ibid., p. 178.

^69^ Ramasamy. *Periyar Kalanjiyam (31).* p. 44.

^70^ Ramasamy. *Periyar Kalanjiyam* (22). pp. 109-110.

^71^ Ibid., p. 102.

^72^ Uma Chakravarti. 2018. *Gendering Caste: Through a Feminist Lens.* New Delhi: Sage, p. 20.

^73^ Ramasamy. *Periyar Kalanjiyam* (22). p. 102.

^74^ Periyar E.V. Ramasamy. 2007. *Periyar Kalanjiyam (23): Pennurimai Pagam 4*. Chennai: Periyar Suyamariyadhai Prachara Niruvanam, p. 22.

^75^ M.S.S. Pandian. 1993. “'Denationalising' the Past: 'Nation' in E V Ramasamy's Political Discourse.” *Economic and Political Weekly*, Vol. 28, No. 42. pp. 2282–2287.

^76^ Gayatri Spivak. 1988. "Can the Subaltern Speak?" In *Marxism and the Interpretation of Culture*. Eds. Cary Nelson & Lawrence Grossberg. Illinois: University of Illinois Press, p. 299.

^77^ Periyar E.V. Ramasamy. 2007. *Periyar Kalanjiyam (24): Pennurimai Pagam 5*. Chennai: Periyar Suyamariyadhai Prachara Niruvanam, p. 130.

^78^ Partha Chatterjee. 1993. *The Nation and its Fragments: Colonial and Postcolonial Histories.* Princeton, NJ: Princeton University Press, p. 117.

^79^ Ibid., p. 121.

^80^ Ibid., p. 131

^81^ Ibid., p. 132.

^82^ Chakravarti. *Gendering Caste,* pp. 120–127.

^83^ V. Geetha. 2018. “Caste, Gender and Spatial Politics.” In *Gender, Caste and the Imagination of Equality.* Ed. Anupama Rao. New Delhi: Women Unlimited, p. 93.

^84^ Periyar viewed the measures taken against the veil in Kemalist Turkey to be liberating for Muslim women. See Ramasamy. *Periyar Kalanjiyam (24),* p. 173. His contemporary, the iconic anti-caste Dalit leader Ambedkar, also attacked the “regressive” social attitudes of the Indian Muslims and argued that “Indian Muslims would be benefitted if they took a leaf out of Kemal’s book.” See Dhananjay Keer. 2019. *Dr. Babasaheb Ambedkar: Life and Mission.* (Fifth Edition). Mumbai: Popular Prakashan, p. 223

^85^ Anandhi. “Women’s Question in the Dravidian Movement”, p. 32.

^86^ Kausalya Sankar. 2017. “Love Will Triumph When Honour Killings Are Prevented. Caste Will Be Annihilated When Love Triumphs.” *The Wire.* 07 December. https://thewire.in/caste/love-will-triumph-honour-killings-prevented-caste-will-annihilated-love-triumphs [Accessed: 07 April 2020]

^87^ Living Smile Vidya & Gee Imaan Semmalar. 2018. “Transphobia is a Kind of Brahminism.” In *Gender, Caste and the Imagination of Equality.* Ed. Anupama Rao. New Delhi: Women Unlimited, pp. 69-70.

