# Supplementary material for: Radical freedom: Periyar and women
Source: Open Res Eur. 2021 Mar 24;1:6. [Version 1] doi: 10.12688/openreseurope.13131.1 (PMC10445885; doi:10.12688/openreseurope.13131.1)
Supplement: Peer review history

# Peer review: Reviewer response for version 1

**DOI:** 10.21956/openreseurope.14201.r26550

**Published:** 2021-06-14

**Authors:** Vandana Swami

**Affiliations:** 1 School of Development, Azim Premji University, Bengaluru, India

The essay ‘Radical Freedom: Periyar and Women’ is a mediation on questions of feminism and the possibilities of women’s complete emancipation that might create conditions for their emergence as free and equal members of society, as envisaged by Periyar. As the author rightly notes, while Periyar wrote voluminously on questions concerning women and feminism, their writings are not as well recognized as his critiques of caste and religion. In bringing these writings to prominence, the author brings renewed attention to an issue of pressing significance to India as well as other societies – that is, gender justice, women’s social oppression and unequal status. A parallel concern in the essay is to put the feminist writings of Periyar in comparison with the work of Shulamith Firestone, whose well known work ‘The Dialectic of Sex’, raised many controversies since it got published in 1970. While documenting some similarities in their work, the reason for comparison, according to the author, is to ‘place the works of the lesser known and marginalized Periyar in global debates on sexual freedom’. A brief review of contemporary Indian feminist scholars writings on caste and gender questions Anandhi, Uma Chakravarti, Sharmila Rege, V Geetha, Sarah Hodges and Anupama Rao is also undertaken, and serves as a useful context to highlight the vexed nature of problems that Periyar was addressing with respect to gender, sexuality and women in India.

 Anandhi’s underlines Periyar’s general observation that while chastity and marriage were key partriarchal institutions of control, the same could be said for how patriarchy had influenced language and literature. V Geetha notes the importance of the Self Respect Movement in providing an alternative form of identity to feminists and women at large who believe that the idea of comradeship should inform a radical female subjectivity. Similarly, Hodges documents the impact of the Self Respect Movement in transforming the family and domestic spheres in Tamil Nadu. Anupama Rao brings out the centrality of caste masculinity over ideals of gender equality even in Dalit politics and notes the weak articulation of the women’s movement and gender issues in movements and spaces before and after Periyar. With the help of this review and the underscoring of his views by leading feminist scholars, the author establishes the significance of Periyar’s strong views on women’s position and gender injustice in Indian and particularly Tamil society. Needless to say, Periyar faced immense hostility and criticism from Tamil nationalists for his progressive outlook at a time where social conservatism dominated and fiercely guarded women’s relegation and confinement into the family and the performance of domestic duties, particularly childbearing and childrearing, as her central and only roles.

 Almost five decades later, the same issues of women’s oppression thinking about ways to end these oppressions were taken up in earnest by second wave feminist Shulamith Firestone. The author discusses her work in some detail, particularly for drawing a contrast with the writings of Periyar. Like him, Firestone also emphasized the liberation of women from childbearing and childrearing roles as the very pivot for social and sexual freedom. In fact, considering that she saw pregnancy as ‘barbaric’ and something responsible for ill health and even death of many women, Firestone even proposed artificial and technologically assisted reproductive mechanisms that would unburden women completely from this ‘barbaric’ biological experience, thereby eliminating male privilege, the stranglehold of the biological family and the ‘sex distinction’ itself.

 While Periyar did not articulate a technological answer like Firestone to end the ‘slave like’ condition of women in childbearing and childrearing, he certainly advocated for contraception, and felt that ‘biological callings’ need not be met only within the confines of a marriage. In that sense, the author notes that Periyar proffered not only a bold and radical sexual freedom for women, but also provided a substantial critique of the notion of love and its disciplining within the confines of the institution of marriage. As the author rightfully states, ‘disciplined and idealized notions of love chained women to their oppression and caste’.

 Taken together, the author has presented a valuable analysis of Periyar’s ideas on women’s freedom, their rights, sexual and social self-determination. Given the paucity of work that has looked at this aspect of Periyar’s writings, this is an important contribution in itself, and also for its re-centering attention on contemporary issues like honor killings, domestic violence, caste atrocities and an overall patriarchal insubordination that continues to mark the life of women in India, despite impressive gains made by women’s movements. To enhance the critical potential of this essay, it is suggested that the author consider the following points:

- The author could consider modifying the second half of the title to some extent. ‘Periyar and Women’ sounds a little odd and problematic, as it would sound odd if the title were to be ‘Periyar and Men’. I suggest that the author consider something like ‘Periyar and the Women’s Question’, or something along those lines. 
- While a comparison of the writings of Periyar and Firestone is interesting, there could be a greater fleshing out of both the reasons and grounds for the kind of comparison that he is making. If the objective is to bring Periyar into global discussions of gender, sexuality and womens emancipation, then a deeper consideration of the motivations, situations, contexts and struggles of Periyar should perhaps frame this inquiry because by the time that Periyar is writing, the anti-colonial struggle against the British in India is very active and the women’s question is being debated by many nationalist leaders. While this aspect is brought in nicely through the work of Partha Chatterjee, it may also strengthen the essay further if the author could locate women’s writings and women’s own responses to Periyar’s position at that moment. Was there any wider dialogue that took place, and in what way did it shape the struggle and questions? It would be useful to hear from those narratives.
- Towards the end of the essay, the author mentions the influence of Periyar on Kausalya, a victim of the atrocity of honor killings and on Living Smile Vidya, a queer rights activist and performance artist in Tamil Nadu as an index of the continuing impact of his thought. While doing so, the author might additionally consider looking for a wider social resonance of Periyar’s thoughts on the women’s question, caste and gender concerns. For instance, what is the contemporary status of the Self Respect Movement and to what extent is it supporting and promoting equal marriages between couples today? What is the sociological reach of the radical potential of what Periyar has to say about Tamil society? Is it only to be found among certain castes or classes, or is it more composite? Has it had any impact on middle and upper classes and the elite Brahmanical groups?
- The author might offer a more critical evaluation of both Periyar and Firestone’s views on the ways and means by which women’s insubordination is to be brought to an end. Firestone’s views in this regard have been read as particularly problematic because of her evisceration of the essential femininity of a woman and the consignment of the power of biological reproduction at the altar of technology. Instead of seeing pregnancy as barbaric, are there ways to see it as empowering for a woman and her social role? Even if one is critical of the family, marriage and love, as Periyar was, what kind of changes would be needed for a greater acceptance and mobilization of both men and women, regardless of caste and class, to self transform themselves to create a gender just society that honors women? In other words, how should these radical ideas be translated for them to become widely discussed and adopted? Where does the putting together of Periyar and Firestone’s views lead us today, if we are looking at India and the possibilities of change?
- A greater clarity could be established on the use of the terms ‘sex’ and ‘gender’ that sometimes appear interchangeable.

Is the argument persuasive and supported by appropriate evidence?

Partly

Is the work clearly and cogently presented?

Yes

Is the topic of the essay discussed accurately in the context of the current literature?

Yes

Does the essay contribute to the cultural, historical, social understanding of the field?

Yes

Reviewer Expertise:

NA

I confirm that I have read this submission and believe that I have an appropriate level of expertise to confirm that it is of an acceptable scientific standard.

# Peer review: Reviewer response for version 1

**DOI:** 10.21956/openreseurope.14201.r26774

**Published:** 2021-05-17

**Authors:** Sowmya Dechamma C. C, Barath Nataraj

**Affiliations:** 1 Department of Journalism & Mass Communication, North Eastern-Hill University Shillong 793022, Meghalaya, India; 2 Centre for Comparative Literature, University of Hyderabad, Hyderabad, India

**Article Review**

**Radical Freedom: Periyar and Women**

 The paper titled “Radical Freedom: Periyar and women” by Karthick Ram Manoharan is an addition to the subaltern feminist scholarship in India. It is an in-depth reading of Periyar’s book *Woman Enslaved, *published in 1934, which is a collection of ten essays that he wrote on the women’s question between 1926 and 1931. The author also brings out his thoughts on Shulamith Firestone’s *The Dialectic of Sex*, a classical feminist text.

**Summary of the article**

 This paper is an effort towards a comparative reading between Shulamith Firestone’s *The Dialectic of Sex *and Periyar’s book, *Women Enslaved.* The importance of this paper lies in the way it has charted out the origins of feminist thoughts of Periyar and to place his writings in the context of global debates on sexual freedom.

 The article begins with tracing the existing debates and scholarship on the women’s question and gender in the context of Tamil Nadu. The author discussed the works of Anandhi and Velayutham which puts forth the need for knowledge generated from the margins as there are ‘limitations in theory itself in dealing with diversities and subalternity’. Then it proceeds to build on the works produced by feminist academics such as Uma Chakravarti, Sharmila Rege and Anupama Rao to narrate their stances on issues of caste and Gender and their intersections.

 The next section of the article discussed at length about Periyar’s approach to women’s question. The author here argues that for Periyar “it was grounded not only in a rights based discourse, but also in a freedom-based discourse; not just freedom from patriarchy, but also sexual freedom in a radically libertarian sense”. He draws few arguments of Firestone on the lines of sexual freedom to point out the familiarity of Periyar’s position on the institution of marriage and family, which according to him is the most oppressive structure for women. As the author points out, for Firestone ‘the heart of women’s opposition is her childbearing and child rearing roles’ and once can see similar opinion voiced out by Periyar when he states pregnancy as “positively harmful to the autonomy of women’ and blamed it for being the root cause of women’s frequent illness, aging before time and premature death.”

 The author’s also demonstrates Periyar’s stance in women’s question which is a radical departure from that of the Indian Nationalists as well as the local Tamil Nationalists. Periyar envisioned an egalitarian society that is rooted in gender equality and freedom of women to exert their agency on their body and sexuality, whereas the nationalist movement and the anti-caste movement did not heed attention to the gender question. This argument of the author is thoroughly evident in the way he rendered an account of Periyar’s thought on the question of women and gender concerns.

**Evaluation of the article**

 The article is relevant as it also reflects on the grim reality of ‘honor Killings’ which has been prevalent among the dominant backward castes as well as the Dalit communities of Tamil Nadu. Alongside the concept of arranged marriage, honor killings are viewed by the caste society to regulate control over individual sexuality and caste purity. The author’s argument on the re-emergence of Periyar thoughts as vital force to combat the casteist forces is evident, in the transformation of Kausalya as an anti-caste intellectual towards the cause of social Justice and annihilation of caste.

 The author could have tried to bring in a section which focuses on how the different feminists groups in India, especially Dalitbahujan feminists from Tamil Nadu like Bama, Sivagami, or Salma, have engaged with Periyar’s feminist ideals. This combined with a few queer narratives would have provided insights into the contemporary relevance of Periyar works and also be informative on the ground realities of how alliances among different marginal communities are negotiated, unlike the highly individualist ideas of sexual freedom that the article focusses on. Karthick’s work does provide the much needed impetus on an on-going debate about sexual freedom. This work adds to the existing narratives of diverse feminist scholarship as well, especially in India where talking about sexual freedom is still in its nascent stages

**Some Suggestions**

 We agree with the other reviewer on many counts including the question of how one can use oral narratives, other means of understanding women’s life-stories other than the ones based on written narratives, within Periyarist politics.

1. There is a slight lack of clarity in the usage of the terms ‘sex’ and ‘gender’, especially in the beginning of the essay. Some consistency and elaboration may help from the author’s own perspective and in relation to Firestone and Periyar’s perspectives.
2. Both in Firestone and Periyar, there is an emphasis on ‘biological’ liberation and its connection to the economic liberation of women. We would like to see how these would actually change the politics of power that interconnects other spheres of our lives as well.
3. From what we understand, “Pativrata’, ‘Stridharma’ are concepts that are not all pervasive (Sati as well). These were and are located in certain caste practices which then assumed a pan-Indian nature during nationalist movement. This means, there is a necessity to locate ‘chastity’ in its caste and other histories. Also, in a subtle way, by essentializing caste values, practices and attributes (prescribed to women) of Brahmin community as something that spilled onto the Dalit-Bahujan castes as well, the author seems to treading close to the Binary of Brahmin and Non-Brahmin. This needs to be contextualized.
4. Although Firestone and Periyar advocate similar politics, what the author misses is this – that despite his critique of West, Periyar saw certain practices of West as more enabling to women. BUT, these are the very same liberal practices of West that Firestone critiqued fifty years later. How can we then justify the comparison between Firestone and Periyar?
5. Periyar advocates a sexual practice that is in tune with human “biological calling” while at the same time, both Periyar and Firestone argue against a ‘naturalized’ biological determinism. The contradiction here between ‘nature’ / ‘natural’, ‘biological’ need to be addressed.
6. We feel the conclusion needs some elaboration and theorization of Periyarist modernity and what exactly is ‘radical freedom’. How did Periyar envision a modernity that emphasized on Liberty, Equality, and Fraternity (and Justice) – the bedrocks what is known as modernity? Was this idea of modernity and radical freedom totally around the question of women? How did Periyar’s engagement with caste and its intersections with gender figure in his idea of freedom? Did freedom mean a very individualist notion of liberation or is there an understanding of mutual respect (along with self-respect), and well-being of people around us? How do these ‘free’ individuals form a community? Is a new community envisioned?

Is the argument persuasive and supported by appropriate evidence?

Partly

Is the work clearly and cogently presented?

Yes

Is the topic of the essay discussed accurately in the context of the current literature?

Yes

Does the essay contribute to the cultural, historical, social understanding of the field?

Yes

Reviewer Expertise:

Sowmya Dechamma C C: Comparative Indian Literature and Cultural Discourses in Contemporary India, Literatures of India, Translation Studies, Minority Languages and Cultural Discourse, Kodava performative cultures. Barath N: Print History, Media and Culture Studies, Development Studies.

We confirm that we have read this submission and believe that we have an appropriate level of expertise to confirm that it is of an acceptable scientific standard.

# Peer review: Reviewer response for version 1

**DOI:** 10.21956/openreseurope.14201.r26773

**Published:** 2021-05-10

**Authors:** Kiran Keshavamurthy

**Affiliations:** 1 IIT Guwahati (Indian Institute of Technology Guwahati), Guwahati, India

Dear Author, I have the following comments and observations on your article. 

1. The term 'brahminical' needs to historicized and not dogmatised to represent everything that the modern progressive mind abhors. Here it is being used (by Periyar) as a hollow signifier that does not capture its complex historical religious and cultural vicissitudes especially in terms of the complex negotiations between Vedic Brahminism, Shaivism, Buddhism and Jainism. There is also a gap between pre-modern tensions and exchanges between Shaivism and Buddhism and 19th century colonial consolidations and codifications of Hinduism and Hindu identity that needs to be addressed. There is a disjuncture between their textual prescriptions of caste endogamy (Manu, Yagnavalkya) and actual experiences of casteism that will not (entirely) conform to textual prescriptions. So I don't think there is a simple imitation or mimicry of upper caste norms ('Sanskritization') that are then filtered down the hierarchy. There are local variations that constitute a multitude of responses to social and sexual norms in terms of challenges, appropriations, exceptions, inter-caste intimacies and transgressions. Chastity, for instance, may not be a concern for everyone. This diversity of responses needs to be considered if one has to think of caste and sexuality as complex, dynamic and socially and historically contingent correlates. 
2. Although Firestone's argument that technologically mediated pregnancies would enable the possibility of female freedom is a powerful argument that enables the possibility of female freedom, it is necessary to firstly, relate the institution of compulsory motherhood to the forces of market capitalism if women truly desire emancipation. This would entail transforming the patriarchal family from a moral to an economic unit where men and women have equal livelihood opportunities and share financial and domestic responsibilities. Secondly, does motherhood have to be an inherently oppressive thing even when it is voluntary in a society where both men and women and transgenders are potential caregivers? If Firestone endorsed artificial pregnancies, Periyar advocated the use of contraceptives. Do they both agree that motherhood should be ultimately done away with? Thirdly, is there any room in Firestone and to a lesser extent in Periyar's thought, for imagining alternative intimacies between and among men and women that are not patriarchal and misogynistic and may involve voluntary childrearing? Here, I am also wondering if there is a possibility of imagining polyamorous and queer relationships that could potentially challenge patriarchal sexual norms. This needs to be clarified as there are points where the author refers to Periyar's emphasis on universal chastity while at other points, Periyar seems to suggest that men and women can have multiple partners. Does marriage have to be a contract between two individuals, a man and a woman and is chastity for Periyar a lauded universal value or not?
3. Is love for Periyar a purely private emotion or can it form the social basis for collective wellbeing? Did he make a distinction between romantic and other forms of love? Again, here the author is requested to be consistent in his representation of Periyar's views. Or if there is an apparent contradiction in Periyar's views, is he being strategic and responding to particular situations? If so, what are the conditions that produce these particular articulations and how does Periyar justify his contrasting views? 
4. Periyar's opinion of the devadasis is a little inconclusive. He believed they should not be exploited and commodified but then what was the alternative? Marriage? Or a more 'respectable' profession? Did he think all devadasis were nothing more than sex workers or if not, was the mere exposure of the female performer to the male gaze in exchange for money/kind sufficient to merit banning? Did he think sex work should be banned because it was morally degrading or economically exploitative and was it because he thought everyone should be chaste? Was marriage even as a secular contract necessary or could (two or more) people live together without the bonds of marriage or choose to remain single? 
5. How effective was the Self-Respect Marriage in breaking down caste barriers between Dalits and dominant castes? 
6. I feel both Periyar and Firestone are solely driven by certain social and political exigencies that prevent them from addressing the nuances, ambiguities and subtleties of various aesthetic forms of expression like religion, art and literature. While I am not suggesting that they should uncritically embraced, it is equally important to pay close attention to the different ways in which men and women (and all other genders) have drawn from various forms of religious and literary practices to (affectively) mobilize people and negotiate with structures of power even in small and relatively insignificant ways, which in turn has made the present possible. The (affective) history of the present is necessary in order to appreciate the present in all its contradictions and complexities instead of merely accepting or rejecting it. 

*I would also like to include folk performances and oral traditions of storytelling along with literary practices.

Is the argument persuasive and supported by appropriate evidence?

Partly

Is the work clearly and cogently presented?

Yes

Is the topic of the essay discussed accurately in the context of the current literature?

Yes

Does the essay contribute to the cultural, historical, social understanding of the field?

Partly

Reviewer Expertise:

Caste, gender and sexuality studies, modern Indian literatures.

I confirm that I have read this submission and believe that I have an appropriate level of expertise to confirm that it is of an acceptable scientific standard, however I have significant reservations, as outlined above.
